# Impact of *Salmonella* Control Programmes in Poultry on Human *Salmonellosis* Burden in Greece

**DOI:** 10.3390/antibiotics10020121

**Published:** 2021-01-28

**Authors:** Myrsini Tzani, Georgia Mandilara, Joana Gomes Dias, Theologia Sideroglou, Anthi Chrysostomou, Kassiani Mellou

**Affiliations:** 1 European Programme for Intervention Epidemiology Training (EPIET), European Centre for Disease Prevention and Control (ECDC), 169 73 Stockholm, Sweden; 2 Department of Foodborne and Waterborne Diseases, Hellenic National Public Health Organisation (EODY), 3-5 Agrafon Str., 15123 Marousi-Attiki, Greece; t.sideroglou@eody.gov.gr (T.S.); a.chrysostomou@eody.gov.gr (A.C.); 3 National Reference Centre for Salmonella, Department of Public Health Policy, School of Public Health, University of West Attica, 196 Alexandras Ave., 11521 Athens, Greece; gmandilara@uniwa.gr; 4 European Centre for Disease Prevention and Control (ECDC), Gustav III:s Boulevard 40, 169 73 Solna, Sweden; joana.gomes.dias@ecdc.europa.eu

**Keywords:** *salmonellosis*, National Salmonella Control Programmes, interrupted time series analysis, public health

## Abstract

Since 2008, veterinary authorities in Greece have implemented national control programmes (NSCPs) targeting *S. Enteritidis* (SE) and *S. Typhimurium* (ST) in poultry. We assessed the effect of the programs on the reported number of human isolates. Using monthly data for 2006–2017, we defined two groups (SE, ST) and one control group with serotypes unrelated to poultry or eggs. For SE we also analysed data for 2006–2015 due to a multi-county SE outbreak in 2016. We performed an interrupted time series analysis and used a negative binominal regression model. For both SE and ST, there was no significant trend of the isolation rate before or after NSCPs’ introduction. After the NSCPs’ introduction there was an increasing rate (IRR: 1.005, 95% CI: 1.001–1.008) for control serotypes and a decreasing one for SE (IRR: 0.990, 95% CI: 0.986–0.995) (for 2009 to 2015 analysis). From 2006 to 2017, NSCPs had a statistically significant impact on the number of SE isolates that decreased by 49% (IRR:0.511, 95% CI: 0.353–0.739). No impact was shown on the number of ST (*p*-value = 0.741) and control isolates (*p* = 0.069). As a conclusion, NSCP’s implementation was associated with decreased SE isolates and overall burden of salmonellosis; however further measures aiming at human salmonellosis due to ST, should be considered.

## 1. Introduction

Non-typhoidal salmonellosis is caused by bacteria of genus *Salmonella* spp. which is one of the most frequently isolated foodborne pathogens, accounting for 93.8 million foodborne illnesses and 155,000 deaths per year [1]. To date, over 2600 *Salmonella* serotypes have been identified and more than half of them belong to *Salmonella enterica* subsp. *enterica* [2].

In the EU, over 90,000 salmonellosis cases are recorded every year making salmonellosis the second most frequently reported zoonotic disease after *Campylobacter* spp. infection [3]. It has been estimated that the overall annual economic burden of human salmonellosis in Europe is as high as three billion euros [3]. The three most commonly reported *Salmonella* serotypes, belonging to *Salmonella enterica* subsp. *enterica,* are Enteritidis, Typhimurium and the monophasic variant of Typhimurium with the antigenic formula 1,4,[5],12:i:- (referred as *S. Enteritidis*, *S. Typhimurium* and monophasic *S. Typhimurium* hereafter). The risk of infection is mostly associated with the consumption of contaminated eggs, pig and poultry meat [3].

To protect consumers from *Salmonella* spp., the EU has adopted an integrated approach to food safety from “farm to fork” that includes the annual monitoring of salmonellosis notification rate in humans, the control of the pathogen in specific animal species and the production of food free of *Salmonella* [4,5,6,7,8,9,10,11]. The control of the pathogen mainly focuses at poultry meat and eggs. Regulation (EC) No 2160/2003 sets targets for the reduction of targeted salmonella serotypes in flocks of breeding hens, laying hens, broilers, breeding turkeys and fattening turkeys [6,7,8,9,10]. The targets are set on two serotypes (*S.* Enteritidis and *S. Typhimurium*, including monophasic *S. Typhimurium*), except for breeding hens for which also *S.* Hadar, *S.* Virchow and *S.* Infantis are also targeted. In order to achieve the targets, Member States have introduced National Salmonella Control Programmes (NSCPs) in poultry populations. NSCPs include enhanced surveillance on the basis of specific sampling protocols according to EU legislation, vaccination against *S.* Enteritidis and *S. Typhimurium* in poultry, implementation of strict biosecurity measures, restriction of movements and destruction or heat treatment of infected birds and eggs, in the event of the detection of targeted *Salmonella* serotypes in a flock of poultry [7,8,9,10,12].

In Greece, 10,321 salmonellosis cases were reported from 2004 to 2019 with a mean annual notification rate of 5.9 cases per 100,000 population [13]. *S. Enteritidis*, *S. Typhimurium* and monophasic *S. Typhimurium* accounted for 80% of the total isolated serotypes in the country [13]. NSCPs were implemented for the first time in 2007 in breeders [14]. In 2008, their implementation expanded to layers, in 2009 to broilers and since 2010, the programmes have also been covering breeding and fattening turkeys [14].

The aim of this study was to evaluate the impact of NSCPs in poultry breeding on human salmonellosis reported number of cases.

## 2. Results

### 2.1. Descriptive Data

From 2006 to 2017, 5,432 human *Salmonella* spp. isolates were serotyped; 42% (2260) were *S. Enteritidis*, 13% (681) *S. Typhimurium*, 5% (247) monophasic *S. Typhimurium* and 40% (2244) other *Salmonella* serotypes. The mean number of human *Salmonella* spp. isolates per year was 453 (min: 241, max: 630).

**Figure 1:**
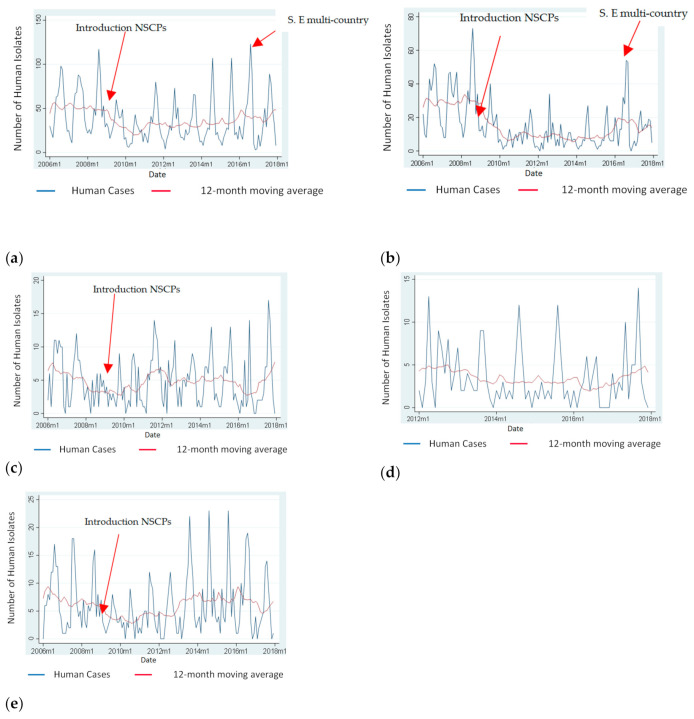
Monthly number of *Salmonella* spp., *S. Enteritidis*, *S. Typhimurium*, monophasic *S. Typhimurium* and “Control *Salmonella* serotypes” of human isolates and 12-month moving average.(**a**) Monthly cases attributed to *Salmonella* spp. and 12-month moving average, Greece, 2006–2017; (**b**) Monthly cases attributed to *S. Enteritidis* and 12-month moving average, Greece, 2006–2017; (**c**) Monthly cases attributed to *S. Typhimurium* and 12-month moving average, Greece, 2006–2017; (**d**) Monthly cases attributed to monophasic *S. Typhimurium* and 12-month moving average, Greece, 2012–2017 (Data on monophasic *S. Typhimurium* were only available since 2012 and the introduction of the NSCPs was much earlier in 2009, therefore there is no arrow in panel (**d**); (**e**) Monthly cases attributed to “Control *Salmonella* serotypes” (serotypes detected from human cases between 2004 and 2017 but were either not detected or rarely detected, maximum of two times each, in poultry) and 12-month moving average, Greece, 2006–2017.

Figure 1 depicts the monthly distribution of *Salmonella* spp., *S. Enteritidis*, *S. Typhimurium*, monophasic *S. Typhimurium* and “Control *Salmonella* serotypes” of human isolates for the period 2006 to 2017 and the annual trend of these serotypes using moving averages. According to the Figure 1, there was a decrease in the total number of *Salmonella* cases, *S. Enteritidis* cases, and cases due to “Control *Salmonella* serotypes” after the implementation of NSCPs in 2008. However, the number of *Salmonella* cases due to the above-mentioned serotypes started to increase again in different time periods for different serotypes. *S. Enteritidis* increased sharply in 2016 and decreased again in 2017.

From 2006 to 2017 the trend of the number of all *Salmonella* serotypes, and of *S. Enteritidis* isolates were statistically significantly decreasing while there was no significant trend for the number of *S. Typhimurium*, monophasic *S. Typhimurium*, and “Control *Salmonella* serotypes” (Table 1).

### 2.2. Results of the Interrupted Time Series Analysis

During the study period 2006–2017, there was no evidence of significant trends in the number of *S. Enteritidis* isolates both, before, as well after, the introduction of the NSCPs. However, the programs had a statistically significant impact on the total number of *S. Enteritidis* human isolates and resulted in a statistically significant decrease of their total number by 49% (IRR: 0.511, 95% CI: 0.353–0.739).

When analysis for 2006–2015 was performed there was no trend of the number of *S.* **Enteritidis** isolates before the introduction of the intervention, however, afterwards there was a statistically significantly decreasing trend and the reported number of *S. Enteritidis* isolates decreased by 0.95% per month on average (IRR: 0.990, 95% CI: 0.986–0.995). The introduction of the NSCPs resulted in a statistically significant decrease by 47% (IRR: 0.526, 95% CI: 0.384–0.720) of the total number of *S. Enteritidis* isolates.

For *S. Typhimurium* we did not find a significant trend both before as well after the introduction of the NSCPs. Additionally, there was no evidence that the implementation of the intervention had an impact on the total number of *S. Typhimurium* isolates.

Finally, regarding the “Control *Salmonella* serotypes”, before the introduction of the intervention there was no significant change in the reported monthly number of those isolates, while after the introduction of the intervention there was an increase by 0.45% on average per month (IRR: 1.004, 95%CI: 1.001–1.008).

Table 2 summarises the results of the interrupted time series analysis for the evaluation of the impact of the introduction of the NSCPs on the trend and the total number of *S. Enteritidis*, *S. Typhimurium* and “Control *Salmonella* serotypes” isolates.

**Figure 2:**
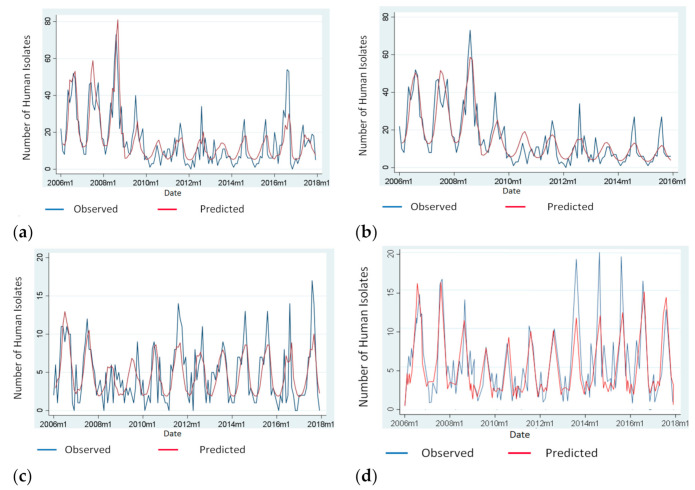
Number of observed and predicted human *Salmonella* isolates, Greece, 2006–2017; (**a**) Number of observed and predicted human *S. Enteritidis* isolates according to the model used, Greece, 2006–2017; (**b**) Number of observed and predicted human *S. Enteritidis* isolates according to the model used, Greece, 2006–2015 (For *S. Enteritidis*, a sensitivity analysis was also performed for both periods 2006–2017 and 2006–2015. The reason was that in 2016 there was an excess in the number of *S. Enteritidis* cases in Greece probably attributed to the multi-country *S. Enteritidis* outbreak related to the consumption of infected Polish eggs); (**c**) Number of observed and predicted human *S. Typhi-murium* isolates according to the model used, Greece, 2006–2017; (**d**) Number of observed and predicted human isolates attributed to “Control *Salmonella* serotypes” (serotypes detected from human cases between 2004 and 2017, but were either not detected or rarely detected, the maximum of two times each, in poultry) according to the model used, Greece, 2006–2017.

Figure 2 illustrates the distribution of *Salmonella* isolates due to: (**a**) *S. Enteritidis* (2006–2017), (**b**) *S. Enteritidis* (2006–2015), (**c**) *S. Typhimurium*, (**d**) “Control *Salmonella* serotypes” over time and the predicted number of isolates based to the model used.

## 3. Discussion

This is the first study in Greece evaluating the impact of NSCPs in poultry breeding on human salmonellosis reported number of cases.

Based on descriptive analysis, the number of human cases attributed to all serotypes, to *S. Enteritidis*, to *S. Typhimurium* and to “Control *Salmonella* serotypes” was decreased after the implementation of the NSCPs in 2008. However, after this initial decrease, the number of cases remained stable or increased for different time periods depending on the serotype.

Overall, the results of the interrupted time series analysis support that the implementation of the NSCPs was followed by a reduction of the number of the total *S. Enteritidis* isolates in humans. This decrease resulted in the reduction of the total number of *Salmonella* isolates in humans. Similarly, a reduction in the human salmonellosis cases was also reported between 2005 and 2013 in several EU countries following the coordinated approach implemented at EU level for the control of salmonella in poultry [14]. However, from 2014 to 2018, the trend in most of the member states became stable [14].

The aforementioned decrease was to be expected as according to EFSA, eggs and poultry meat are the main sources of transmission for *S. Enteritidis* and in Greece the NSCPs are only implemented in poultry populations [14]. Therefore, the implementation of control measures against this serotype in poultry populations is important. The positive impact of the implementation of NSCPs on human salmonellosis has also been demonstrated in other member states and at European level [14,15,16].

On the other hand, NSCPs did not have an impact on the number of *S. Typhimurium* isolates, although Greece has achieved the EU targets in both layers and broilers and the prevalence of *S. Typhimurium* in both poultry species is very low [17,18]. Therefore, we can assume that NSCPs did not affect the trend and the number of *S. Typhimurium* isolates and that identified cases were probably attributed to other sources of infection, such as swine animals and their products [14]. This is the reason why other countries have extended control programs to other susceptible animal species based on the results of a cost benefit analysis conducted at a national level [16,18,19].

According to our analysis, the number of reported monophasic *S. Typhimurium* 1,4 [5],12: i: - isolates remained at about the same levels between 2012–2016 and increased in 2017. Although this serotype has been detected from fattening turkeys in Greece [17,18], turkeys cannot be considered an important source of human monophasic *S. Typhimurium* infection due to the small size of the turkeys’ population in the country [17]. Taking also into account reports on the investigation of *S. Typhimurium* outbreaks, we can consider that pork meat and its products are important sources of monophasic *S. Typhimurium* infection in Greece [13].

Finally, the increasing trend of the number of “Control *Salmonella* serotypes” after the programmes’ implementation and the fact that programs did not have an impact on the number of “Control *Salmonella* serotypes” further supports our previous conclusions as we can assume that the reduction in the number of *S. Enteritidis* cases was not affected by a factor that had influenced *Salmonella* infection overall. On the other hand, the increased number of cases might suggest that other potential sources of infectivity exist and demonstrates the need for health education of the public regarding the prevention of salmonellosis infection, good hygiene and cooking practices [20]. There is no doubt that educating the public will further enhance the positive impact of the NSCPs on the protection of public health from the targeted Salmonella serotypes. Finally, it is possible that the eradication of *S*.*Enteritidis* may leave a niche for other serotypes to fill [21].

A primary limitation of our study was that the absence of MLVA and WGS data in Greece did not allow us to differentiate outbreak-related cases from sporadic ones. We considered this limitation especially important during the European *S.* Enteritidis multi-country outbreak in 2016 and 2017, the largest ever documented *S.* Enteritidis outbreak in Europe, which was attributed to Polish-eggs originating from infected flocks [13]. This is why sensitivity analysis was also performed for *S.* Enteritidis excluding 2016–2017 [22].

Another limitation regards the use of laboratory data on serotypes as proxy of the number of salmonellosis cases in humans, although we consider that this had a small impact on our results as the system was overall stable [23].

In conclusion, data support that the implementation of the NSCPs in poultry populations was followed by a decreased number of human salmonellosis cases. Programmes should continue, in order to keep meeting the EU targets, but for an effective public health program, the implementation of measures is needed in other animal species, as well as in all steps of food production (from farm to fork).

## 4. Materials and Methods

We extracted data from the National Reference Laboratory for *Salmonella* and *Shigella* (NRLSS) database in Greece for the period 2006–2017. The 2018–2019 data were excluded from the analysis as non-comparable with previous years due to changes in the operation of the reference laboratory. The NRLSS receives human *Salmonella* strains from the collaborating hospital laboratories across the country for serotyping and antimicrobial susceptibility testing. This system is universal but not mandatory and specimens sent at NRLSS are a proxy of the number of diagnosed salmonellosis cases at medical services of the country [23].

### 4.1. Descriptive Analysis

Outbreak-related cases were excluded from the analyses. Reported cases were classified as outbreak-related when the results of investigation indicated an epidemiological link to other reported salmonellosis cases with the same serotype. Time-series were plotted by month and summary statistics of the number of isolates per month and year were calculated to describe the distributions on possible outliers and missing data. Proportions of the different targeted *Salmonella* serotypes were also calculated and the distribution of the different serotypes over time (month and year) was plotted. Moreover, data were assessed for stationarity by checking for constant mean using a linear regression model and constant variance using a mean versus variance plot and autocorrelation (autocorrelation and partial autocorrelation). Normality was assessed using a Shapiro-Wilk normality test. To describe the trend and seasonality of the time series we used moving averages. Windows size 6 and 12 were used to highlight seasonality, and annual trend, respectively. Additional cyclical patterns were also assessed using spectral analysis.

Analysis was conducted separately for (a) *Salmonella* spp. serotypes, (b) *S. Enteritidis*, (c) *S. Typhimurium*, (d) monophasic *S. Typhimurium*, (e) “control *Salmonella* serotypes”.

“Control *Salmonella* serotypes” were those detected from human cases between 2004 and 2017, but were, either not detected or rarely detected (maximum of two times each) in poultry based on the data of the surveillance system for animal salmonellosis of the Greek Ministry of Rural Development and Food for 2011–2017 [17].

### 4.2. Interrupted Time Series Analysis

To assess the impact of the intervention (introduction of NSCPs) on the reported number of human salmonellosis cases, we used interrupted time series analysis and looked for changes after the intervention in important parameters of the model; intercept, slope and cyclical patterns. We defined intervention (*S. Enteritidis* and *S. Typhimurium*) and control series.

Monophasic *S. Typhimurium* isolates were excluded as they have started to be recorded separately from *S. Typhimurium* only since 2012, after NSCPs had been launched.

Control group (“Control *Salmonella* serotypes”) was used to test if the results for the targeted *Salmonella* serotypes had been affected by factors other than NSCPs’ implementation. This group was used as a “baseline” of the trend of the reported number of human salmonellosis cases in Greece.

The study period for the interrupted time series analysis was 2006 to 2017. However, for *S.* Enteritidis, a sensitivity analysis was also performed for both periods 2006–2017 and 2006–2015. The reason was that in 2016 there was an excess in the number of *S.* Enteritidis cases in Greece probably attributed to the multi-country *S. Enteritidis* outbreak related to the consumption of infected Polish eggs [14].

Analysis was conducted separately for each series. We set the time of introduction of the intervention in January 2009, despite the fact that the implementation of the NSCPs in breeders, layers started in 2007, and 2008 respectively, as it takes some time for the NSCPs to produce results, namely reduction of the prevalence of the targeted *Salmonella* serotypes, in poultry species producing animal products for human consumption.

Poisson and negative binomial regression models were used to study the impact of the intervention on the number and trend of the recorded isolates. The selection of the final model for the evaluation of the intervention’s impact was based on the residual analysis performed for these two models and was a negative binominal regression model which included trend and a sine wave with a 12-month period to adjust estimates for secular and cyclical trends, the intervention (a binominal variable), the interaction between trend and intervention and one lag to count for autocorrelation.

IRR values and the respective CIs were calculated. In all cases, *p*-values < 0.05 were considered statistically significant.

Residuals of each model were plotted against the model and checked for stationarity, autocorrelation and normality. Additionally, residuals were tested with Portmanteau test to check if they were statistically significantly similar to white noise.

Analysis was performed in Stata v16.1. [24].

## Figures and Tables

**Table 1:** Trends of the number of *Salmonella* spp., *S*. *Enteritidis*, *S. Typhimurium*, monophasic *S. Typhimurium* and “Control *Salmonella* serotypes” of human isolates, Greece, 2006–2017.

Salmonella Serotype	Trend
*Salmonella* spp.	Statistically significantly decreasing (IRR: 0.996, *p*-value < 0.001, 95% CI: 0.995–0.998)
*S. Enteritidis*	Statistically significantly decreasing (IRR: 0.992, *p*-value < 0.001, 95% CI: 0.990–0.994)
*S. Typhimurium*	No evidence for significant trend (IRR: 0.998, *p*-value = 0.287, 95% CI: 0.996–1.001)
Monophasic *S. Typhimurium*	No evidence for significant trend (IRR: 0.992, *p*-value = 0.134, 95% CI: 0.983–1.002)
“Control *Salmonella* serotypes” ^1^	No evidence for significant trend (IRR: 0.999, *p*-value = 0.834, 95% CI: 0.997–1.002)

^1^ serotypes detected from human cases between 2004 and 2017 but were either not detected or rarely detected, maximum of two times each, in poultry.

**Table 2:** Results of the evaluation of the impact of the introduction of the National Salmonella Control Programmes (NSCPs) on the trend and total number of *S*. *Enteritidis*, *S*. *Typhimurium* and “Control *Salmonella* serotypes” of human isolates.

*Salmonella* Serotypes	Trend of Salmonellosis before the NSCPs’ Implementation	Trend of Salmonellosis after the NSCPs’ Implementation	Impact of the NSCPs on the Total Number of *Salmonella* Isolates
*S. Enteritidis* (2006–2017)	No evidence for significant trend (IRR = 1.001, *p*-value = 0.886, 95% CI = 0.983–1.019)	No evidence for significant trend (IRR = 1.000, *p*-value = 0.804, 95% CI = 0.997–1.003)	49% decrease (IRR: 0.511, *p*-value < 0.001, 95% CI: 0.353–0.739)
*S. Enteritidis* (2006–2015) ^2^	No evidence for significant trend (IRR = 1.002, *p*-value = 0.781, 95% CI = 0.987–1.017)	Statistically significant decreasing trend (IRR: 0.990, *p*-value < 0.001, 95% CI: 0.986–0.995)	47% decrease (IRR: 0.526, *p*-value < 0.001, 95% CI: 0.384–0.720)
*S*. *Typhimurium*	No evidence for significant trend (IRR = 0.981, *p*-value = 0.077, 95% CI = 0.962–1.001)	No evidence for significant trend (IRR = 0.999, *p*-value = 0.994, 95% CI = 0.996–1.003)	No evidence for statistically significant impact (IRR = 0.941, *p*-value = 0.741, 95% CI = 0.660–1.343)
“Control *Salmonella* serotypes” ^3^	No evidence for significant trend (IRR = 0.990, *p*-value = 0.226, 95% CI = 0.974–1.006	Statistically significant increasing trend (IRR:1.004, *p*-value = 0.011, 95% CI: 1.001–1.008)	No evidence for statistically significant impact (IRR: 0.0001, *p*-value = 0.069, 95% CI: 0.000–1.987)

^2^ For *S*. *Enteritidis*, a sensitivity analysis was also performed for both periods 2006–2017 and 2006–2015. The reason was that in 2016 there was an excess in the number of *S*. *Enteritidis* cases in Greece probably attributed to the multi-country *S*. *Enteritidis* outbreak related to the consumption of infected Polish eggs. ^3^ serotypes detected from human cases between 2004 and 2017 but were either not detected or rarely detected, maximum of two times each, in poultry.

## Data Availability

The data presented in this study are available on request from the corresponding author. The data are publicly available at the official website of the National Reference Laboratory for *Salmonella*, *Shigella* and other enteropathogens at http://www.mednet.gr/whonet/, but only until 2016.
